# Internet-Based Cognitive Behavioral Therapy and Virtual Reality Exposure Therapy for Social Anxiety Disorder: Protocol for a Randomized Controlled Trial in Hong Kong

**DOI:** 10.2196/48437

**Published:** 2023-09-14

**Authors:** Jia-Yan Pan

**Affiliations:** 1 Department of Social Work Hong Kong Baptist University Hong Kong China (Hong Kong)

**Keywords:** internet-based cognitive behavioral therapy, virtual reality exposure therapy, social anxiety disorder, Chinese, CBT, iCBT, internet, virtual reality, anxiety, social phobia, mental disorder, mental health, quality of life, treatment, cognitive behavioral therapy, psychological intervention

## Abstract

**Background:**

Social anxiety disorder (SAD), also known as social phobia, is one of the most common mental disorders worldwide. In Hong Kong, the prevalence of SAD is high, but its treatment rate is low. SAD has immense impact on academic or work performance, social life, career development, and quality of life. One of the most effective treatments for SAD is cognitive behavioral therapy (CBT), with internet-based CBT (iCBT) and virtual reality exposure therapy (VRET) showing promise in treating SAD. However, internet interventions are underdeveloped in Chinese communities including Hong Kong.

**Objective:**

This study aims to develop an iCBT program that includes VRET, called “Ease Anxiety in Social Event Online” (Ease Online), for Hong Kong adults with SAD in a randomized controlled trial.

**Methods:**

The 14-week Ease Online program is a guided self-help iCBT program with a blended mode of service delivery. The program comprises 9 web-based modules and 5 individual counseling sessions (including 2 VRET sessions) conducted remotely or face-to-face with a therapist to provide therapist support, as guided iCBT shows superior effects than unguided iCBT. Other program components include therapist feedback on assignments, internal messages, forums, client portfolios, web-based questionnaires, reminders, and web-based bookings. The program can be accessed either through a mobile app or program website through a PC with an internet connection. The participants are openly recruited and screened using a questionnaire and through an intake interview. Eligible participants are randomized by placing them into a web-based iCBT group, app-based iCBT group, or a waitlist control (WLC) group. Participants in the WLC group are assigned to the app-based program upon completion of the service of the 2 experimental groups. Measurements of social anxiety, depression and anxiety symptoms, psychological distress, automatic thoughts, and quality of life are administered at pretest, posttest, and 3- and 6-month follow-ups. Multivariate ANOVA with repeated measures will be performed to determine the intervention effectiveness on the continuous variables over time.

**Results:**

Participant recruitment commenced in January 2021. As of February 2023, a total of 1811 individuals applied for the Ease Online program. In total, 401 intake interviews have been completed, and 329 eligible participants have joined the program, among whom 166 have completed the service. Data collection is still ongoing, which is expected to be completed in March 2024.

**Conclusions:**

This study is the first of its kind in combining iCBT and VRET for the treatment of SAD in Hong Kong. At a theoretical level, this study contributes to the development and evaluation of internet-based psychological interventions in Hong Kong. At a practical level, the Ease Online program may serve as an alternative service option for SAD clients in Hong Kong if proven effective.

**Trial Registration:**

ClinicalTrials.gov NCT04995913; https://clinicaltrials.gov/study/NCT04995913

**International Registered Report Identifier (IRRID):**

DERR1-10.2196/48437

## Introduction

### Background

Social anxiety symptoms have increased significantly during the COVID-19 pandemic [1]. Social anxiety disorder (SAD) is one of the most common mental health problems, yet few seek treatment [2]. Individuals with SAD experience excessive fear and anxiety during social interactions, which is disproportionate to the social situation itself. SAD often involves negative evaluation and rejection by others and a constant fear of embarrassment or humiliation [3]. Therefore, individuals with SAD often avoid social interactions, which may have detrimental impacts on most areas of life, including their career, education, social relationships, and mental health [4]. Social anxiety usually emerges during performance situations (such as job interviews, public presentations, or attending a meeting) and interpersonal situations (such as greeting someone, drinking and eating in public, taking part in social gatherings, or shopping). In Hong Kong, the 12-month prevalence rate of SAD is reported to be 3.2% [5], which translates into an estimated 230,000 cases. However, only 8.7% of people with SAD in Hong Kong had previously sought treatment [5].

People with SAD face certain treatment barriers that prevent them from seeking mental health services, including fear of judgement, uncertainty of the resources for receiving help, and skepticism regarding anxiety disorders [6]. In Hong Kong, the barriers also include the social and self-stigmas of mental illnesses, long waiting lists for public health care, and exorbitant costs of private treatment. However, people with SAD consider the internet as a good social outlet, which not only helps them to alleviate their fear during general social interactions but also meets their social needs [7].

Cognitive behavioral therapy (CBT) is the first-line treatment for SAD [8]. Clark and Wells [9] proposed a cognitive model of social anxiety that considers social cognition, safety behaviors, self-focused attention, and somatic symptoms as the maintaining factors of social anxiety. Thus, the effective treatment components of CBT for SAD primarily include exposure to fearful conditions, cognitive restructuring, attentional bias modification, and relaxation and social skills training [10]. Telemental health services, including internet-based CBT (iCBT) and virtual reality exposure therapy (VRET), have become a worldwide trend for the treatment of mental health problems, particularly SAD, which can reduce the therapist’s time required for each client [11]. For example, the average time spent in VRET is 8 minutes per session [12], and the therapist’s time in iCBT can be saved by more than half for the same level of reduction in social anxiety [13].

### iCBT for SAD

iCBT is used to deliver CBT on the web or through a mobile app. In a recent meta-analysis study, Andrews et al [14] showed that there are no significant differences between iCBT and face-to-face CBT in their efficacy for treating anxiety and depressive disorders, thus indicating equivalent overall effects elicited by both types of treatments. Their study also lends support to the effectiveness, acceptability, and practicality of iCBT for treating anxiety disorders. A number of randomized controlled trials (RCTs) have demonstrated the effectiveness of guided iCBT in reducing general and social anxieties, avoidance, depression, and dysfunctional thinking, as well as improving the quality of life and functioning of people with SAD [15-19]. Guided iCBT programs for SAD have shown promising diagnostic response rates [20] with complete and partial recovery rates of 34.2% and 36.8%, respectively, upon completion of the treatment [19]. The positive treatment effects are maintained at the 6-month [19], 1-year [15], 2-year [21], and 4-year [22] follow-ups. Therapist contact is reportedly a significant predictor of intervention outcomes in guided iCBT programs for SAD [23]. Therapist support is usually provided via telephone, email, videoconferences, SMS text messages, and web-based discussion forums [17,24].

The intervention skills in the various iCBT programs for SAD generally include psychoeducation, cognitive restructuring, behavioral experiments, exposure and reality testing, modifying attention bias, social skills training, developing healthy lifestyles, problem-solving, and preventing relapses [15,20,21]. The presentation formats of the iCBT program involve text, images, audio, video, text entry fields, animation, comic slides, e-books, etc [24]. Interventions include monitoring moods, performing exposure exercises and homework assignments, writing a web-based diary, answering essay questions, participating in user forums, and receiving emails or text message reminders, and awards [17,18].

### VRET for SAD

One of the central components of CBT for SAD is exposure therapy in which clients are exposed to social stimuli that cause anxiety while eliminating safety behaviors so that they learn and understand that the anticipated negative consequences are ill-founded [3]. Exposure therapy can activate and modify anxious responses by having individuals confront the fear stimuli and via habituation and extinction learning, respectively, in which the fear stimuli then cease to provoke anxious responses or render them less threatening. Given that in vivo exposure therapy in real life is not frequently offered, VRET has become an important alternative for reproducing social situations [3]. Computer graphics, body tracking devices, visual displays, and other sensory input devices are integrated to immerse clients in a computer-generated virtual environment [25]. As virtual reality (VR) can elicit moderate levels of sense of presence and anxiety during virtual social interactions [26], VRET has become a promising application for treating SAD.

Meta-analyses have consistently found a significantly large effect size for VRET compared to a waitlist or psychological placebo conditions for SAD treatment, and no significant difference in effect size between VRET and in vivo or imaginal exposure therapy [27,28], thus indicating that VRET is effective for SAD treatment with effects comparable to those of traditional exposure therapy. Specifically, VRET is effective in reducing social anxiety symptoms, such as fear and avoidance, fear of negative evaluation, self-reported public speaking anxiety, catastrophic belief expectancy, probability and cost biases, distress, perceived stress, and depressive symptoms, and increasing the length of speech time, perceived performance quality, and confidence [2,16,29,30]. The positive effects are maintained at the 3-month, 6-month, 12-month, 5.5-year, and 6-year follow-ups [29,31,32]. More than half of treated individuals no longer meet the diagnostic criteria for SAD (54%) and self-reported to be “very much” or “much” improved (68%) 6 years after treatment [31]. Thus, VRET is considered an efficacious alternative to in vivo exposure therapy [27].

Both psychological and physiological indicators are measured in VRET studies. The commonly used psychological indicators include self-reported anxiety and fear, distress, and self-confidence [33,34]. As anxiety often emerges in the form of physical symptoms, VRET studies have measured physiological responses, such as blood oxygen level [35] and heart rate [36].

### This Study

Technology-assisted CBT programs have great potential to extend mental health services to people with social anxiety who are unwilling or unable to seek face-to-face therapy [23]. However, these programs are underdeveloped in Chinese societies, such as Hong Kong. This study has, therefore, developed an iCBT program with VRET, called “Ease Anxiety in Social Event Online” (Ease Online), for Hong Kong adults with SAD in an RCT.

## Methods

### Participants

To be included in the study, participants should be Hong Kong residents who (1) are fluent in Cantonese, (2) are aged between the 18 and 70 years, (3) obtained a score of 24 or higher on the Chinese version of the Social Phobia Inventory (SPIN) [37], (4) have a social anxiety disorder as assessed by the Structured Clinical Interview for disorders listed in the Diagnostic and Statistical Manual of Mental Disorders, 4th Edition [38], (5) have not received any psychological treatment at the time of registration for participation in this study, (6) have access to a computer or smartphone with an internet connection, and (7) have a valid email address. We will exclude individuals who have (1) severe depression, with a score above 30 on the Beck Depression Inventory-II (BDI-II) [39]; (2) suicidal risk in the past 3 months (score of item 9 on the BDI-II is “2” or “3” and assessed to have suicidal risk in the intake interview); and (3) a severe psychiatric condition (eg, bipolar disorder, schizophrenia, or personality disorder) diagnosed by a psychiatrist or clinical psychologist.

### Sample Size

The sample size was calculated by using a computer program called G*Power (version 3.1.5; Universität Düsseldorf). The statistical method used was a multivariate ANOVA with repeated measures (within-between interactions). The effect size was assumed to be 0.40 (large) [40]. The number of groups was 3 (2 experimental groups and 1 control group) and 7 measures applied (please see the *Outcome Measures* section). The α value was set at .01. The calculations resulted in a minimum sample size of 139. This study aims at recruiting 330 eligible participants, with 110 in each experimental group and the waitlist control (WLC) group, respectively.

### Participant Recruitment and Screening

Participants were recruited from the general population on an ongoing basis through various means, such as advertisements on social media (Facebook and Instagram) and Google, press conferences, posters, and referrals from local nongovernmental organizations. Interested participants were asked to complete a screening questionnaire that includes the Chinese version of the Beck Anxiety Inventory (C-BAI), BDI-II, and Chinese version of the SPIN on the program website [41]. Initially qualified participants were invited for a 1.5-hour intake interview in person or via Zoom with a psychological counselor or an experienced social worker for further assessment of suitability for participation (including assessment of suicidal risk and social anxiety disorder based on the diagnostic criteria of the Structured Clinical Interview for disorders listed in the Diagnostic and Statistical Manual of Mental Disorders, 4th Edition [38]). Marginal cases were discussed in a case meeting (which involved author J-YP, an intake worker, and a program therapist) for participation eligibility. A project assistant contacted the eligible participants to reconfirm their desire to participate before randomization took place. High-risk individuals with severe depression or suicidal risk (or both) identified at the screening stage were referred to the local mental health service sectors for further diagnosis and treatment with their consent.

### Research Design

Although deterioration in a control condition can take place [42], Steinert et al [43] conducted a meta-analysis and found only small changes in the WLC groups during short-term wait periods for clients with SAD in RCTs. Thus, this study adopts a WLC group design to conduct a 3-arm RCT as shown in Table 1.

**Figure 1:**
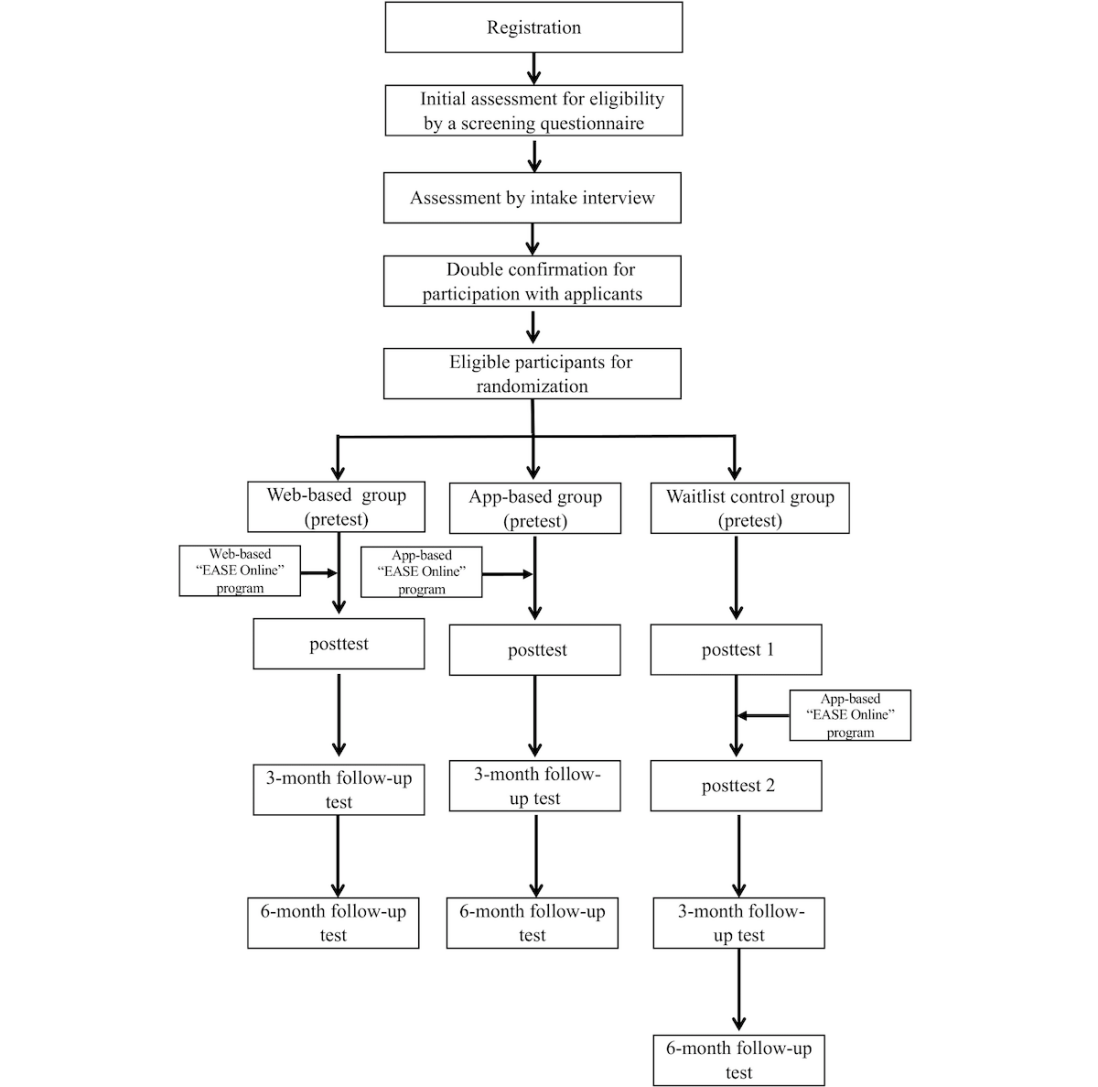
Flowchart for participant recruitment in this study. Ease Online: Ease Anxiety in Social Event Online.

There were 2 experimental groups that joined either the web-based or app-based iCBT program (Ease Online). The WLC group received the app-based treatment immediately after the 2 experimental groups completed the program. All 3 groups completed the same web-based questionnaire at pretest, posttest, and 3- and 6-month follow-ups. The WLC group completed the questionnaire one more time at posttest after the 2 experimental groups completed the program. To increase the submission rate of questionnaire, participants were given 2 more weeks to submit an email questionnaire after the submission deadline in the program system. The RCT was conducted in cohorts for implementation feasibility. Specifically, in each cohort, around 30 qualified participants were randomized into one of the 3 groups through computer-generated random numbers, with approximately 10 in each group. The program evaluators were blinded to the allocation of the participants. The participant recruitment flowchart is shown in Figure 1.

**Table 1:** Participant group design.

Groups	Research design						
Web-based	O^a^	X^b^	O		O	O	N/A^c^
App-based	O	X	O		O	O	N/A
Waitlist control	O		O	X	O	O	O

^a^O: test.

^b^X: intervention.

^c^N/A: not applicable.

### Intervention: the Ease Online Program

The Ease Online program is 14 weeks in duration. The program adopts a blended mode of service delivery as guided self-help iCBT has demonstrated lower attrition than nonguided self-help iCBT [18], and therapist contact has been found to be a significant predictor of retention and outcomes [23]. The program consists of 9 web-based modules and 5 individual counseling sessions (including 2 VRET sessions). The program contents were developed on the basis of the CBT model developed by Beck [44] and CBT delivered to Chinese clients by Wong [45] and Pan and Zhuang [46]. Specifically, a variety of cognitive behavioral skills for coping with social anxiety are introduced in the 9 web-based modules, each with a specific theme. The themes of the nine web-based modules are (1) recognizing your social anxiety responses, (2) identifying automatic thoughts and cognitive distortions, (3) self-talk and positive statements, (4) behavioral experiment, (5) fear hierarchy, (6) exposure therapy, (7) identifying cognitive rules, (8) relaxing dysfunctional cognitive rules, and (9) saying bye-bye to social anxiety. The CBT skills are briefed and debriefed by using an animation video and demonstrated through 4 case videos. Each web-based module starts with a mood check for social anxiety in the past week and ends with a session review and an assignment that helps clients to apply the learned skills to cope with their own social anxiety. Other components of the program include therapist feedback on assignments, internal messages, forums, client portfolios, web-based questionnaires, reminders, and web-based bookings. An introduction video of the program provides a navigation on all of the program components after the clients log into the system. The client platform has a web-based version in which clients can log into the program website or a mobile app that can be downloaded through Google Play or Apple Store. After being randomized into the program, clients were provided an account to log into the system. Clients who are assigned a PC web account cannot log in through the app and vice versa. All clients were assigned to a therapist who not only provided web-based feedback on their assignments and responded to their private messages, but also offered 5 individual counseling sessions that monitored the progress of the clients and addressed any challenges that the clients encountered when working on the web-based modules. There is also a backstage management platform for the therapist, which offers different functions including creating cohorts, managing bookings and forums, providing feedback on client exercises, monitoring client progress, etc. The therapist helped the clients understand and review the module contents and apply the CBT skills into their own situations in 5 individual counseling sessions that were conducted either in person or on Zoom or WhatsApp.

**Figure 2:**
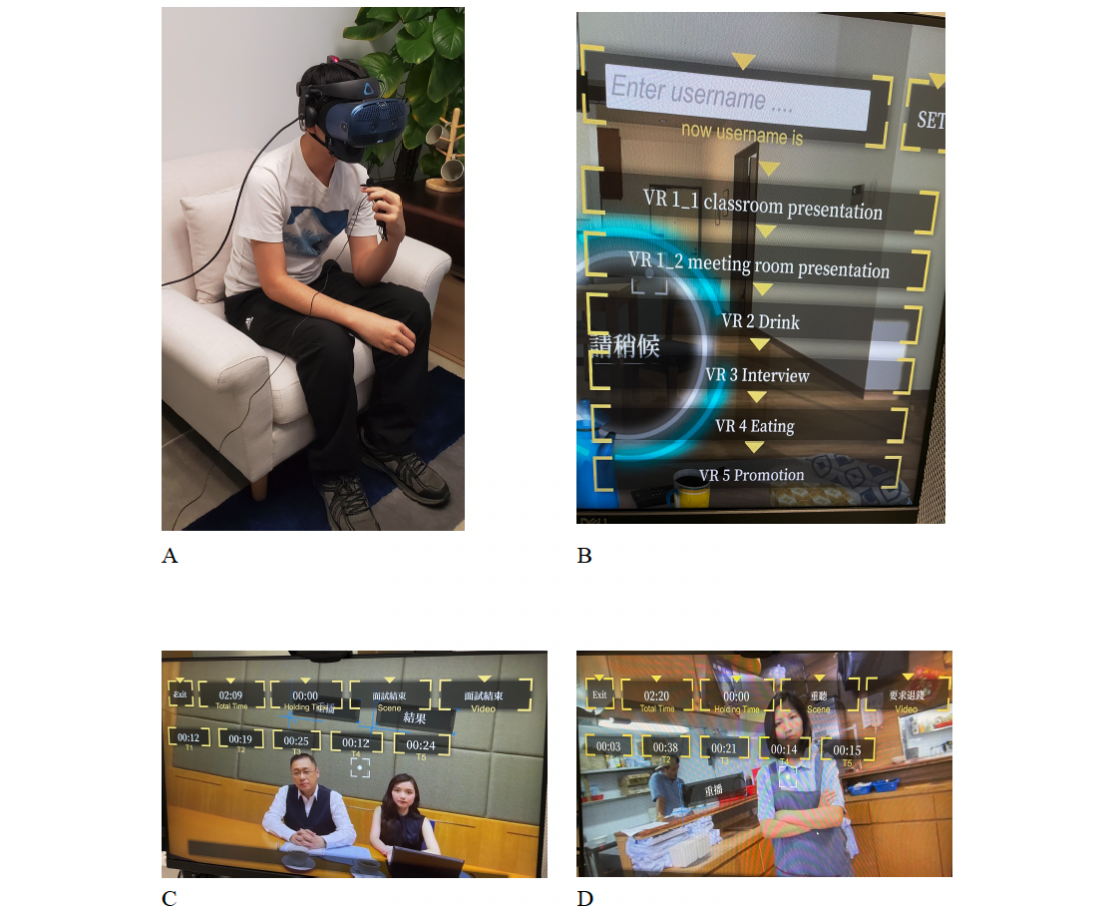
Examples of images in virtual reality exposure therapy. (A) Client wearing a head mounted display. (B) Control panel of the VR system. (C) Virtual scenario of work performance appraisal. (D) Virtual scenario of expressing disagreement. VR: virtual reality.

A VR system for exposure therapy has also been developed for this program, which includes 5 virtual scenarios: (1) interviewing for a job, (2) delivering a presentation, (3) participating in work performance evaluation, (4) expressing disagreement, and (5) drinking in public. The VR system uses an 180° video of real humans. There is a storyline for each VR scenario and a couple of interactions between the client and the VR environment, which are guided by a prerecorded voice-over. The clients wear a head-mounted display and respond by moving their head. The voice responses of the clients are recorded in the VR system for replay. The technologies used for the development of the VR system include voice recording, VR180 stitching, VR game engine development, VR motion tracking on the target point, and an auto-trigger based on the user’s headset direction. An administration control panel has been developed for the therapist to control the VR interaction. In the beginning of each VRET session, the therapist provides a short briefing and asks the client to choose one VR scenario. Then, the client participates in the VRET for no longer than 15 minutes. The therapist then proceeds to debrief the client upon completion of each VRET session and submits a session report on the Ease Online system. Examples of VRET photographs are shown in Figure 2.

The program sessions outlined in Table 2 are released to the client on a weekly basis. The treatment schedule is programmed into the Ease Online system. Progress is recorded as a score when the client completes a task, and a total score is calculated as the total progress. All of the program videos are tailored for Hong Kong people and developed from local cases. The Cantonese language is used in the program. To ensure data security and privacy, 2-factor authentication is adopted when logging in with a password that is preset by the user and a one-time password sent to his/her email address. All of the collected electronic data are encrypted. The program is hosted on the server of the Information and Technology Office of the university where the author is employed. Before the program launched, a user acceptance test was conducted by the author and project staff, and bugs were fixed accordingly by the programmer of this project.

**Table 2:** Structure and content of the Ease Anxiety in Social Event Online program.

Week	Session	Mode	Theme
0	F0	F2F^a^	Intake interview
1	1	Web– or app–based	Recognizing your social anxiety responses
2	2	Web– or app–based	Identifying automatic thoughts and cognitive distortions
3	3	Web– or app–based	Self-talk and positive statements
4	F1	F2F	Counseling session (1)
5	4	Web– or app–based	Behavioral experiment
6	5	Web– or app–based	Fear hierarchy
7	6	Web– or app–based	Exposure therapy
8	F2	F2F	Counseling session (2)
9	F3	F2F	VR^b^ exposure therapy (1)
10	F4	F2F	VR exposure therapy (2)
11	7	Web– or app–based	Identifying cognitive rules
12	8	Web– or app–based	Relaxing dysfunctional cognitive rules
13	9	Web– or app–based	Saying bye-bye to social anxiety
14	F5	F2F	Counseling session (3)

^a^F2F: face-to-face session (in-person or on Zoom/WhatsApp).

^b^VR: virtual reality.

### Therapist Credibility

The therapist for the Ease Online program has a master’s degree in counseling (clinical mental health) and is registered as a certified counselor in both the United States and Canada. She has 9 years of counseling experience in Hong Kong. The intake worker is a registered social worker in Hong Kong who has a bachelor’s and a master’s degree in social work and counseling experience that spans more than 30 years in a variety of settings. The intake interviews were conducted by both the therapist and the intake worker, and all the service and initial screenings were delivered and conducted by the therapist—both are native Cantonese speakers.

### Compliance With the Intervention

Several strategies were adopted to ensure compliance with the intervention: (1) progress reports were completed and submitted by the therapist after each individual counseling session to record the understanding of the clients of the web-based modules and their difficulties in learning them, and applying CBT skills to cope with their own social anxiety; (2) the therapist completed and submitted a progress report in the system after each VRET session to record the session contents, including the VRET experience of the client, skills that the clients have learned to cope with social anxiety, and client feedback; the VRET process was recorded for debriefing in each VRET session; and (3) all the tasks completed by the clients were recorded in the system. A reward score was assigned to clients when they completed a task. Clients were provided a program workbook as an incentive if they completed 80% of the intervention contents.

### Outcome Measures

#### Primary Outcome Measures

The Chinese version of the SPIN [37,47] was used to assess the severity of social phobia symptoms. The measure consists of 17 items with 3 subscales: fear, avoidance, and physiological response. Participants were asked to rate their distress for each symptom during the past week on a 5-point Likert scale that ranges from “0” (not at all) to “4” (very much so). All of the item scores were summed up into a total score that ranged from 0 to 68, with a higher score indicating a higher level of anxiety. Sample items included “fear of others watching,” “avoid talking to others,” and “feeling bothered by blushing.” The α coefficient is .89 for the Chinese version of the SPIN [37].

#### Secondary Outcome Measures

The C-BAI [48,49] was used to measure the cognitive and somatic symptoms of anxiety. Participants were asked to rate 21 items on a 4-point Likert scale. Item scores were summed, with a higher total score indicating a higher level of anxiety. Sample items included “unable to relax” and “heart pounding or racing.” The α coefficient is .95 for the C-BAI [49].

The Chinese version of the BDI-II [39,50] was used to measure depressive symptoms. Participants were asked to rate 21 items on a 4-point Likert scale. The item scores were summed, with a higher total score indicating a higher level of depression. Sample items included “sadness” and “loss of pleasure.” The α coefficient is .86 for the Chinese version of the BDI-II [50].

The Chinese version of the General Health Questionnaire-12 [51] was adopted to evaluate psychological distress. The 0-0-1-1 scoring method was used to calculate the scale score (which ranged from 0 to 12), with higher scores indicating higher levels of psychological distress. The Cronbach α is .87 in a Hong Kong Chinese sample [52].

The Chinese Automatic Thoughts Questionnaire [53] contains 14 items for assessing positive and negative automatic thoughts. Participants were asked to rate the frequency of each thought in the past week on a 5-point Likert scale (which ranged from 1=“not at all” to 5=“all the time”). Item scores were summed up as the 2 subscale scores, with higher scores indicating more positive and negative automatic thoughts, respectively. Sample items included “I am worthless” and “No matter what happens, I know I’ll make it.” The Cronbach α is .83 in a Hong Kong Chinese sample [53].

The Chinese adaptation of the abbreviated version of the World Health Organization Quality of life Scale [54] was used to assess quality of life. This measure consists of 28 items, including 2 items that measure the overall quality of life and 26 items that measure physical and psychological health, social relationships, and the environment. Each item was rated on a 5-point Likert scale that ranged from 1=“very dissatisfied” to 5=“very satisfied,” with a higher score indicating better quality of life. The Cronbach α ranges from .59 to .78 and test-retest reliability ranges from 0.72 to 0.83 for this scale [54].

The Chinese version of the Internalized Stigma of Mental Illness scale [55,56] was used to evaluate the subjective experiences of self-stigma on mental illness. The measure consists of 24 items and 4 subscales: shame or alienation, stereotype endorsement, perceived discrimination, and social withdrawal. Participants were asked to rate items on a 4-point Likert scale that ranged from 1=“strongly disagree” to 4=“strongly agree.” All items were averaged as a scale score, with a higher score indicating a higher level of self-stigma. The Cronbach α is .93 in a Hong Kong Chinese sample [56].

In addition, before and after each VRET session, physical arousal through heart rate and blood oxygen levels were measured with a smart watch that the clients wore on their wrist.

### Ethical Considerations

Ethics approval was granted by the Human Research Ethics Committee (REC/18-19/0241) of the Hong Kong Baptist University. Participation in this study is voluntary and free of charge. Participants could withdraw at any time without any repercussions. High-risk cases identified at the screening stage were referred to local nongovernmental organizations or governmental social service sectors upon the consent of the applicant. Each participant was assigned a code number for identification in the program database to protect their privacy and anonymity.

### Data Analysis

Intent-to-treat analysis, which is the least biased way to estimate an intervention’s effects [57], was used for data analysis. Missing data will be managed using multiple imputation [58]. Multivariate ANOVA with repeated measures will be performed to test the intervention’s effectiveness on continuous variables over time. Time (pretest, posttest, and 3- and 6-month follow-ups), condition (web-based iCBT, app-based iCBT, and WLC), and the interaction of time-by-condition will be specified as the categorical fixed factors. Medication and demographic factors (eg, gender, age, and marital status) will be specified as fixed covariates to control the potential confounding effects. Cohen *d* will be calculated as the index of the effect size both within and between groups.

## Results

The Ease Online program was developed between July 2019 and December 2020 and officially launched in a press conference on January 18, 2021. The principal investigator and project staff have since been involved in promoting the program and recruiting, screening, and referring participants, taking part in intake interviews and case meetings, conducting service delivery and monitoring duties, collecting data, modifying and enhancing the system, providing technical support, responding to enquiries, and other tasks. As of February 2023, a total of 1811 individuals were enrolled in the Ease Online program. A total of 401 intake interviews have been completed, and 329 participants have joined the program, among whom 166 have completed the intervention. A total of 1924 written comments have been provided for client assignments, 604 individual counseling sessions have taken place, and 392 VRET sessions have been conducted. Data collection is still ongoing, which is expected to be completed in March 2024.

## Discussion

### Overview

SAD often has a high prevalence and low treatment rate. People with SAD often underperform and underachieve. Treatment of SAD can not only improve their achievement and quality of life, but also increase productivity in the society as a whole. This study is the first of its kind in combining iCBT and VRET for the treatment of social anxiety in Hong Kong. At a theoretical level, this study will contribute to the development and evaluation of internet-based psychological interventions in Chinese communities. At a practical level, if proven to be effective, this study will provide an alternative e–mental health service option for people in Hong Kong with easy access. In addition, if adopted by local social service sectors, the Ease Online program will fill in a large service gap between the overwhelming service demand by mental health clients and the insufficiency of mental health professionals in Hong Kong.

### Lessons Learnt

In the process of developing the Ease Online program, several lessons have been learnt: (1) considering the extremely low treatment rate of SAD in Hong Kong (8.7%) [5], iCBT serves as a more preferred and accessible treatment option for Hong Kong clients with SAD; flexibility in time and space as well as the web-based format might encourage people with SAD to seek treatment in a way that is less threatening to them. (2) Culturally relevant intervention contents are important. All of the case demonstration videos were tailored for Hong Kong clients with SAD based on local cases, which increases their relevance and resonates with the real-life situations of the clients. The program focuses on working with culturally relevant beliefs related to social anxiety by choosing CBT skills that have been suitable for Chinese clients in previous studies; for example, the “pie chart” technique is used to relax the cognitive rule of “I have to make no mistakes to be a good offspring.” (3) Compared with cartoons or 3D characters, the use of real humans in VR videos may increase the sense of reality and presence in VR situations. (4) Guided self-help iCBT in service delivery, including therapist support in the form of exercise feedback and counseling sessions, is important to help clients to apply the CBT skills learned from the web-based modules to deal with their social anxiety. (5) Compared to text-based iCBT, Hong Kong people prefer video-based iCBT. As Hong Kong people are generally very busy, they may not have time to read long passages of text. Watching short videos that present the key CBT skills with case demonstration is more acceptable to Hong Kong clients. (6) Participant screening using a screening questionnaire and through intake interviews and case meetings ensures eligibility and screening out of high-risk cases and referring other resources to them. (7) Both the web-based platform and the mobile app are developed as computers and smartphones are widely used in Hong Kong. (8) Aside from psychological assessment, the use of physiological assessments, such as heart rate and blood oxygen levels, in VRET can provide more objective evaluations.

### Limitations

There are several limitations of this study that should be considered. First, only completed tasks are recorded in the system, but there are no records of therapist time and use time of the client in the system. User time can be recorded in the system in future research to calculate the average therapist time spent and the amount of time for which the clients use the system. Second, the number of missing data at posttest and follow-up is quite high—this may be related to the busy lifestyle in Hong Kong and nature of free services provided in this study, although various means including email and phone calls have been used to remind clients to complete the web-based questionnaires after the intervention. Different approaches need to be used in future research to increase the response rate of postintervention assessments. Third, although several strategies are adopted ensuring compliance with the intervention, future research may include independent ratings by certified cognitive behavioral therapists of a random selection of the progress reports of the individual counseling and VRET sessions as well as assignment feedback. Fourth, participants are mainly recruited via open recruitment, which may cause recruitment difficulties and a high nonresponse rate after enrollment. Collaboration with local social service sectors is suggested in future research for participant recruitment and follow-up with service delivery and data collection. Fifth, in the VR system, 180° videos are produced, which may limit the view of clients in the VR environment. Future research may aim to develop 360° videos for the VRET system. Finally, 5 VR scenarios are developed in this study; future research may aim to develop more VR scenarios to allow clients to choose social situations that are more relevant to them.

## Abbreviations

BDI-II: Beck Depression Inventory-II
C-BAI: Chinese version of the Beck Anxiety Inventory
CBT: cognitive behavioral therapy
Ease Online: Ease Anxiety in Social Event Online
iCBT: internet-based cognitive behavioral therapy
RCT: randomized controlled trial
SAD: social anxiety disorder
SPIN: Social Phobia Inventory
VR: virtual reality
VRET: virtual reality exposure therapy
WLC: waitlist control
